# Supplementary material for: Wound Healing Impairment in Type 2 Diabetes Model of Leptin-Deficient Mice—A Mechanistic Systematic Review
Source: Int J Mol Sci. 2022 Aug 3;23(15):8621. doi: 10.3390/ijms23158621 (PMC9369324; doi:10.3390/ijms23158621)
Supplement: Supplementary file 1 [file ijms-23-08621-s001.zip › ijms-1820743-supplementary.pdf]

Systematic review protocol

PICO

- Population – leptin-deficient mice (db/db or ob/ob) with wounds or ulcers
- Intervention – any
- Comparison – any
- Outcome – any type of wound healing assessment:
  - Macroscopic assessment (wound size measurement)
  - Histological assessment (wound size, inflammation, remodeling, immunohistochemistry etc.)
  - Molecular analyses (gene expression, epigenetics etc.)
- Study design – all except for reviews, letters and editorials, English language only

Draft title: Examining fidelity of leptin-deficient murine models of diabetic wound healing after 35 years of research.

SUMMARY

Context: Murine leptin-deficient model is widely used in wound healing studies. The mechanism underlying wound healing impairment have not been systematically described. Such a review could help understand previous research given the larger context, as well as help plan future research.
Objective: To summarize all data on mechanisms underlying wound healing impairment in leptin-deficient murine model.
Data sources: English articles from MEDLINE, EMBASE, Web of Science and Scopus
Study selection: vide Study design in PICO
Data extraction: Data extraction from articles by AS, followed by a review by WP, using predefined data fields.
Data synthesis: non-applicable
Conclusions: An impressive number of studies relies on db/db or ob/ob mice as T2DM model for wound healing in both basic and applicatory research. Despite their limitations hindering the transnationality of results into the human setting, they remain reliable and widely studied animal models. Their strength is based on a diligently studied mechanisms of wound healing on both local and systemic levels along with satisfactory reflection on pathologies present in human diabetic wounds. Mechanistic studies revealed some degree of resemblance and discrepancy between the animal models and human physiology. With some precaution, especially concerning monogenic nature of the model, they may be further used in preclinical studies of wound healing for incisional and excisional lesions with comorbid diabetes mellitus.

Search engines

Population: (Mice [MeSH] OR “mice” OR Mice, Inbred C57BL[MeSH] OR Mice, Obese [MeSH]) AND ((“lepr” AND “db/db”) OR (“lep” AND “ob/ob”) OR “leptin-deficient” OR “leptin-receptor-deficient” OR “ob/ob” OR “db/db”) NOT human[mh]
Intervention: -
Outcome: wound healing [MeSH] OR “woun*” OR “ulcer*”

Final (Pubmed): (wound healing [MeSH] OR woun* OR ulcer*) AND (Mice [MeSH] OR "mice" OR Mice, Inbred C57BL[MeSH] OR Mice, Obese [MeSH]) AND (("lepr" AND "db/db") OR ("lep" AND "ob/ob") OR "leptin-deficient" OR "leptin-receptor-deficient" OR "ob/ob" OR "db/db") NOT human[mh]
Yield: 279

Embase - ('wound healing'/exp OR 'wound healing' OR (('wound'/exp OR wound) AND ('healing'/exp OR healing)) OR 'woun*' OR 'ulcer*') AND ('db/db mouse'/exp OR 'db/db mouse' OR 'ob/ob mouse'/exp OR 'ob/ob mouse')
Yield: 198

Scopus (Elsevier)

TITLE-ABS-KEY
((woun* OR ulcer*) AND ("mice" AND ("lepr" AND "db/db") OR ("lep" AND "ob/ob") OR "leptin-deficient" OR "leptin-receptor-deficient" OR "ob/ob" OR "db/db")) AND (LIMIT-TO (LANGUAGE , "English")) AND (EXCLUDE(DOCTYPE , "re") OR EXCLUDE (DOCTYPE , "no") OR EXCLUDE (DOCTYPE, "ch")) AND (EXCLUDE (LANGUAGE, "Croatian")) AND (EXCLUDE (SRCTYPE, "k"))
Yield: 398

Web of Science
ALL FIELDS:((woun* OR ulcer*) AND (("mice" OR "murine") AND ("lepr" AND "db/db") OR ("lep" AND "ob/ob") OR "leptin-deficient" OR "leptin-receptor-deficient" OR "ob/ob" OR "db/db"))
Refined by: [excluding] **DOCUMENT TYPES:** (EDITORIAL MATERIAL OR REVIEW OR BOOK CHAPTER OR LETTER) AND **LANGUAGES:** (ENGLISH)
Yield: 558

Total: 1433
After deduplication: 734

Search performed on the 5th of May 2021.

Search strategy and verification

Step 1. Duplicates' removal

Step 2. Screening by title:

- Is the study design appropriate? I.e. is it an original study and not a review, letter, editorial etc. Conference papers should be included!
- Is this an *in vivo* study, in which a wound or ulcer model in db/db or ob/ob mice was used?
- Was there any intervention aimed at accelerating the healing processes in animals? → add to group "Interventions"
- If not, was it a study aiming at examining the fidelity of the model / verifying the model / improving the model? → add to group "model studies"

If at least 3 x "yes" → Study included in step 3
If not certain → Abstract screening
If the answer to any of the first two points is "no" after abstract screening → Study excluded

Step 3.

- Verification of inclusion and exclusion criteria via full text screening

Inclusion criteria:

- Appropriate PICO

Exclusion criteria:

- Inadequate PICO
